# 97448 Biopsychosocial Determinants of Pain Assessment and Management - Medical and Surgical Trainees’ Perspective

**DOI:** 10.1017/cts.2021.730

**Published:** 2021-03-30

**Authors:** Jennifer A Villwock, Barbara Polivka

**Affiliations:** University of Kansas Medical Center

## Abstract

ABSTRACT IMPACT: Better understanding how clinicians make decisions about pain management, particularly since our prior research has demonstrated that opioids prescribed at discharge is the strongest predictor of opioids taken, is critical to decrease high-risk medication prescribing while preserving high-quality care. OBJECTIVES/GOALS: (1) Identify major biological, psychological, and social determinants of medical and surgical residents’ pain management decisions; (2) Determine salient themes regarding the experience of residents in the management of acute and chronic pain METHODS/STUDY POPULATION: Focus groups of internal medicine and general surgery residents at an academic, tertiary care training hospital located in an urban setting were conducted. Due to the COVID-19 pandemic, all focus groups were conducted virtually and occurred during required didactic sessions to facilitate participation. All interviews were recorded and transcribed. Two reviewers independently reviewed and coded the data following the principles of constructivist grounded theory. RESULTS/ANTICIPATED RESULTS: 42 residents participated in ten focus groups ranging in size from two to five individuals. Six themes emerged demonstrating salient BPS factors in pain management decisions: (1) patient and clinician expectations determine what is considered normal/acceptable; (2) inability of pain scales to reliably capture patient pain; (3) desire for more objective methods of pain assessment, while simultaneously recognizing that pain is an inherently subjective experience; (4) difficulty in determine when pain is 'real’ or 'legitimate'; (5) lack of education and protocols regarding pain management; (6) the importance of engaging other services such as acute pain service or nurse educators in complicated situations. Junior residents often expressed doubt in the appropriateness of their approaches and decisions. DISCUSSION/SIGNIFICANCE OF FINDINGS: Surgical and medical trainees routinely treat pain and may struggle, particularly in the early phases of training, to determine if pain levels are appropriate. There is also a lack of education and/or best practices for assessing and managing pain. These areas represent high-value, clinician-focused targets for future interventions to improve care.

